# Reperfusion therapy in acute ischemic stroke: dawn of a new era?

**DOI:** 10.1186/s12883-017-1007-y

**Published:** 2018-01-16

**Authors:** Sonu Bhaskar, Peter Stanwell, Dennis Cordato, John Attia, Christopher Levi

**Affiliations:** 1 Western Sydney University (WSU), School of Medicine, South West Sydney Clinical School, Sydney, NSW 2170 Australia; 2 Liverpool Hospital, Department of Neurology & Neurophysiology, Liverpool, 2170 NSW Australia; 3 The Sydney Partnership for Health, Education, Research & Enterprise (SPHERE), Liverpool, NSW Australia; 4 Stroke & Neurology Research Group, Ingham Institute for Applied Medical Research, 1 Campbell Street, Liverpool, NSW 2170 Australia; 5 School of Medicine, University of New South Wales (UNSW), Sydney, NSW Australia; 6 Department of Neurology, John Hunter Hospital, Newcastle, NSW Australia; 7 Priority Research Centre for Stroke & Brain Injury, Faculty of Health & Medicine, Hunter Medical Research institute (HMRI) and School of Medicine & Public Health, University of Newcastle, Newcastle, NSW Australia; 8 Centre for Clinical Epidemiology & Biostatistics, Hunter Medical Research Institute, University of Newcastle, Newcastle, NSW Australia

**Keywords:** Stroke, Reperfusion therapy, Prognosis, Endovascular treatment, Neurointervention

## Abstract

Following the success of recent endovascular trials, endovascular therapy has emerged as an exciting addition to the arsenal of clinical management of patients with acute ischemic stroke (AIS). In this paper, we present an extensive overview of intravenous and endovascular reperfusion strategies, recent advances in AIS neurointervention, limitations of various treatment paradigms, and provide insights on imaging-guided reperfusion therapies. A roadmap for imaging guided reperfusion treatment workflow in AIS is also proposed. Both systemic thrombolysis and endovascular treatment have been incorporated into the standard of care in stroke therapy. Further research on advanced imaging-based approaches to select appropriate patients, may widen the time-window for patient selection and would contribute immensely to early thrombolytic strategies, better recanalization rates, and improved clinical outcomes.

## Background

An overwhelming number of studies and clinical trials confirm the efficacy of thrombolytic therapy, in a given therapeutic window, in improving the clinical outcome and recovery of acute ischemic stroke (AIS) patients [1–5]. The primary therapeutic goal for patients with AIS is the timely restoration of blood flow to salvageable ischemic brain tissue that is not already infarcted [6]. Reperfusion therapy using thrombolysis, including intravenous (IV) tissue plasminogen activator (tPA) and endovascular interventions such as mechanical thrombectomy (MT), are the only approved treatments for AIS. Both these treatment options have limitations when used as monotherapies. The only pharmaceutical agent approved for the treatment of AIS is IV-rtPA; however, it is not effective in patients with AIS due to large artery occlusion, where the clot burden is very high. In such patients, MT has proven more effective. Currently, the primary criterion for candidate selection in reperfusion is the time from stroke symptom onset. Reperfusion therapy must be administered within a narrow window time of up to 4.5 h after symptom onset for IV-tPA, and up to 6–8 h for endovascular MT. The restriction on IV-tPA treatment beyond 4.5 h disqualifies the majority of stroke patients admitted beyond this time-window (around 85%), thereby drastically limiting the eligible population [7–10].

In this article, we review the literature on the various reperfusion strategies available for AIS patients, and provide insights on potential applications, limitations of various reperfusion strategies and role of imaging in guiding therapy.

### Evolution of reperfusion therapy

**Fig. 1:**
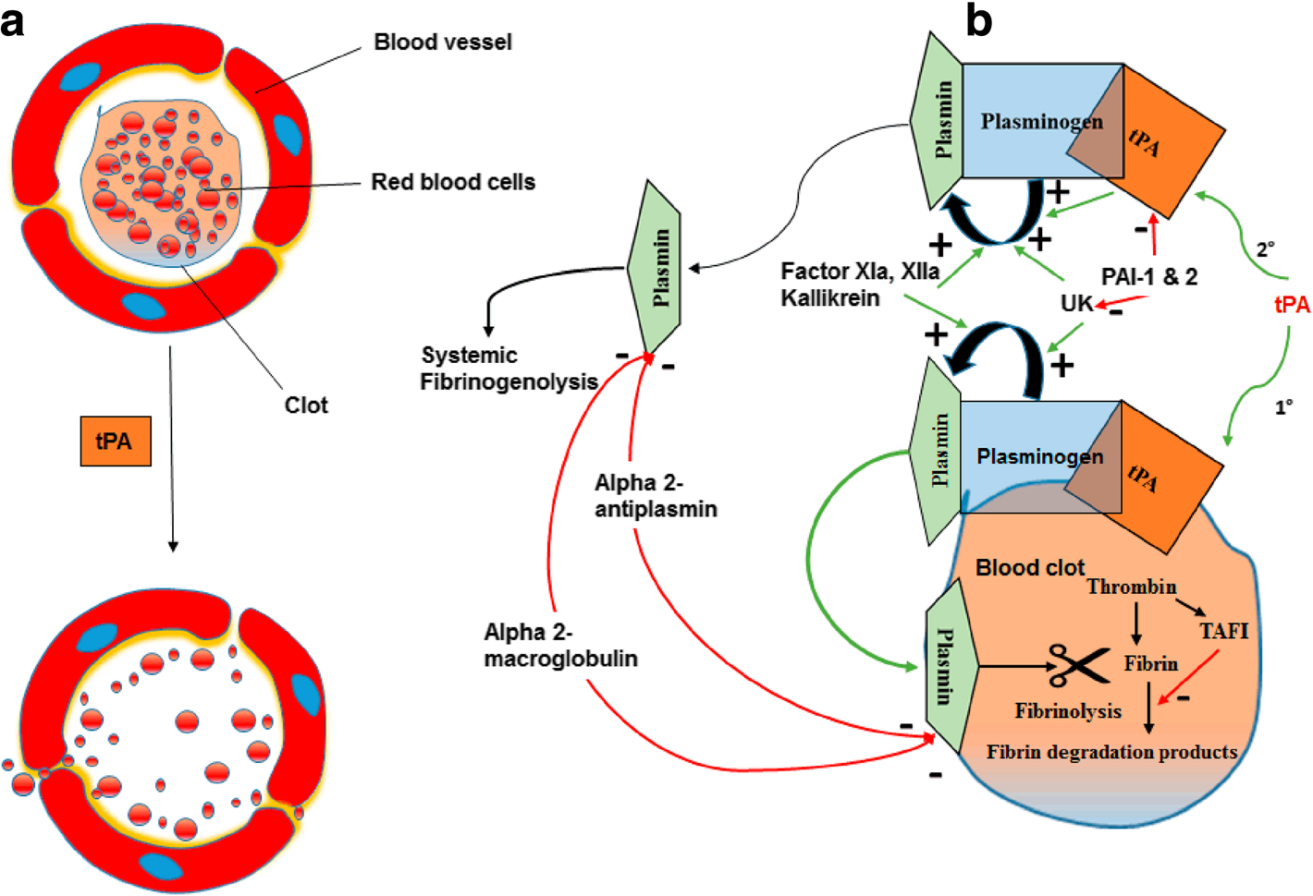
The illustration of the fibrinolytic mechanisms: (**a**) tissue plasminogen activator (tPA) causes breakdown of the clot, and (**b**) detailed mechanism of fibrinolysis. Green arrow denotes activation/stimulation, and the red arrow indicates inhibition. tPA = tissue plasminogen activator; UK = Urokinase; PAI = plasminogen activator inhibitor

Thrombolysis, commonly known as “clot-busting”, is a pharmacological treatment using an infusion of analogues of tPA which leads to the breakdown (lysis) of the culprit blood-clot. Thrombolytic drugs dissolve blood clots by activating a proteolytic enzyme, plasminogen, to plasmin. Fibrin molecules provide the structural scaffold for blood clots, and plasmin cleaves cross-linkages between fibrin molecules. Subsequently, the clot becomes soluble and undergoes further degradation through proteolysis by other enzymes, eventually restoring blood flow. Due to their mechanism of action, thrombolytic drugs are also referred to as “plasminogen activators” or “fibrinolytic drugs”. The three major classes of plasminogen activators are (i) tPA, (ii) streptokinase (SK), and (iii) urokinase (UK). Thrombolytic drugs differ in the mechanism by which they act on fibrin clots. The sequence that leads to the breakdown of the clot or fibrinolysis by tPA is shown in Fig. 1. At first, tPA binds to clot-bound fibrin. This activates fibrin-bound plasminogen from an inactive form to plasmin, the active form. The enzyme plasmin acts on the fibrin mesh, leading to the breakdown of the fibrin scaffold. Subsequently, the dissolution of the fibrin clot produces circulating fibrin fragments called fibrin degradation products. These products prevent the conversion of fibrinogen to fibrin, which slows down clot formation. The liver and kidney, ultimately clear these products, as well as other proteases. Two serine protease inhibitors, namely plasminogen activator inhibitor-1 and 2 (PAI-1 and PAI-2), endogenously inhibit tPA and UK. Alpha 2-antiplasmin and alpha 2-macroglobulin also act as inhibitors of plasmin. Factors XII, XIIa, and Kallikrein stimulate the process of plasmin formation from plasminogen. On the other hand, another factor called thrombin-activatable fibrinolysis inhibitor (TAFI) alters the fibrin to make it more resistant to plasminogen activated by tPA. Efficacy of thrombolytic drugs depends on the size, location, and age of the clot because of the increased density of fibrin cross-linking which make clots more compact and harder to dissolve the older they are.

Some of the known tPA analogues are Alteplase (Activase®; recombinant tPA (rtPA)), Retaplase (Retavase®) and Tenecteplase (TNK-tPA). Alteplase, the most commonly used thrombolytic drug, is a fibrin-selective analogue of tPA administered intravenously or intra-arterially. It is the only Food and Drug Administration (FDA)-approved thrombolytic agent for the treatment of AIS. rtPA causes lysis of fibrin; thereby dissolving the thrombus and resulting in recanalization of the occluded artery [6]. It has a short half-life (~5 min) and is therefore administered as an IV bolus followed by an infusion. Several trials including the National Institute of Neurological Disorders and Stroke (NINDS) [11] and European Collaborative Acute Stroke Study (ECASS) have demonstrated evidence for the benefit of rtPA for a select group of AIS patients. In comparison to rtPA, TNK-tPA has a greater binding affinity for fibrin, higher resistance to inactivation by PAI-1, and a longer half-life.

Urokinase, also known as urinary-type plasminogen activator (uPA), is found in urine. Clinically, UK is used as a thrombolytic drug in conditions such as pulmonary embolism, myocardial infarction, and severe deep venous thrombosis (DVT). In comparison to rtPA, SK and UK lack fibrin specificity and bind equally to circulating plasminogen and clot-bound plasminogen. On binding with plasminogen, SK forms a complex that activates plasmin, triggering a proteolytic cascade. The proteolytic cascade leads to thrombolysis (clot degradation in AIS) or extracellular matrix degradation (e.g., tissue degradation causing tissue invasion and metastasis in tumour malignancy) depending upon the physiological environment. Therefore, SK and UK have less favourable adverse event profiles than rtPA. Thrombolytic drugs can be used as (a) Intravenous, and (b) Intra-arterial. Next, we will discuss the important aspects of these thrombolytic procedures.

### Intravenous thrombolysis

Table 1 gives a detailed overview of clinical trials that evaluated the “time is brain” paradigm. The selection of patients for IV thrombolysis was based mainly on time since stroke onset. All of these trials used non-contrast CT (NCCT) for assessment of parenchymal injury. The exclusion criteria were the presence of a large infarct occupying more than one-third of the MCA territory or causing severe oedema or mass effect visible on NCCT [7].

**Table 1:** List of thrombolytic trials in acute ischemic stroke based on time-window

Trial (n)	Remarks
	TIME WINDOW 0–3 HOURS
NINDS [4, 231] (*n* = 624)	Time window: 0–3 h, 3–6 h Endpoints: A favourable outcome was defined as recovery with minimal or no deficit 3 months after treatment using four outcome measures: the BI ≥95, mRS seven ≤1, Glasgow Outcome Scale 8 of 1, and NIHSS score ≤ 1. Results: Treatment with tPA within 3 h of the onset led to the improved clinical outcome at 3 months, and increase in the incidence of symptomatic ICH.
SITS-MOST [232] (*n* = 6483)	Time window: 0–3 h Endpoints: Primary outcomes were symptomatic (a deterioration in NIHSS score of ≥4) ICH type 2 within 24 h and mortality at 3 months. Functional independence (defined by an mRS score of 0–2 at 90 days) was a secondary outcome. Results: Treatment with Alteplase is safe and effective when used within 3 h of stroke onset.
TESPI [64, 233] (*n* = 248)	Time window: 0–3 h Endpoints: Primary endpoint for efficacy was the disability at day 90, dichotomized as a favourable outcome (mRS 0–2) or unfavourable outcome (mRS 3–6) [64]. The end-point for safety is symptomatic ICH radiologically confirmed on the 22–36 h post-treatment scan combined with neurological deterioration leading to an increase of ≥1 point/s on the NIHSS scale. Results: Higher mortality in patients aged >80 years than younger patients treated with IV-rtPA. No significant differences in symptomatic ICH nor for a favourable outcome. Thrombolytic therapy should not be a priori denied for appropriately selected >80-year old patients [233].
SITS-NEW [234] (*n* = 591)	Time window: 0–3 h Endpoints: Primary endpoints were symptomatic (deterioration in NIHSS score ≥ 4 or death within the first 24 h) intracerebral haemorrhage type 2 22–36 h after the thrombolysis, and mortality at 90 days follow-up. The secondary outcome was functional independence (mRS 0–2) at three-months [234]. Results: IV alteplase is safe and efficient in the treatment of ischaemic stroke in Asian population in congruence to the observations of SITS trials performed on European population.
	TIME WINDOW 3–4.5 HOURS
ECASS-III [11] (*n* = 821)	Time window: 3–4.5 h Endpoints: Primary outcome was a disability at 3 months, assessed by the mRS as either favourable (score of 0 or 1) or unfavourable (score of 2 to 6). Secondary end-points included combined BI and RS, Scandinavian Stroke Scale (SSS) at 90 days, and 30 day mortality. Tertiary outcomes included early neurologic recovery (SSS) and duration of in-hospital stay. Results: Significant benefit of IV-rtPA when administered up to 4·5 h. Symptomatic ICH is significantly more likely with alteplase than with placebo. No difference in mortality between the groups.
CASES [20] (*n* = 1112)	Time window: 3–4.5 h, 0–3 h Endpoints: The primary endpoints were mRS at 90 days, mortality and symptomatic ICH. An mRS 0–1 at 90 days was defined as a favourable outcome. Results: IV alteplase is efficacious in treating AIS patients in 3–4.5 h window; however, there is a tendency towards increased risk of symptomatic ICH in the later time window.
SITS-ISTR [21] (*n* = 23,942)	Time window: 3–4.5 h Endpoints: Primary endpoints were functional independence at 3 months, and incidence of symptomatic ICH.Results: Safety and the functional outcome less favourable after 3 h.
	TIME WINDOW 4.5–6 HOURS
ATLANTIS-B [18] (*n* = 613)	Time window: 3–5 h Endpoints: Primary efficacy endpoint was an excellent neurologic recovery at day 90 (NIHSS ≤1); Secondary endpoints included remarkable recovery on functional outcome measures (BI, mRS, and Glasgow Outcome Scale) at days 30 and 90. Serious adverse events such as symptomatic ICH were also assessed. Results: No significant rtPA benefit on the 90 day efficacy endpoints in patients treated between 3 and 5 h. A significant increase in the risk of symptomatic ICH with treatment using IV-rtPA.
ECASS-II [17] (*n* = 800)	Time window: 0–3 h and 3–6 h Endpoints: The primary outcome was the mRS at 90 days, dichotomised for favourable (score 0–1) and unfavourable (score 2–6) outcome. Results: Trend towards benefit for alteplase, though not statistically significant. Increased risk of symptomatic ICH (8.6% alteplase-group vs. 3.4% placebo-group).
ATLANTIS-A [19] (*n* = 142)	Time window: 0–6 h Endpoints: Primary efficacy endpoints were the number of patients with a decrease of ≥ points on the NIHSS scale at 24 h and day 30, along with infarct volume at day 30. Secondary outcomes included mortality and functional status on the BI and mRS scales at days 30 and 90. Results: Significantly higher proportion of 4-point NIHSS improvement at 24 h for rtPA groups (40%) vs. placebo (21%). The trend reversed at 30 days with more improvement observed in placebo (75%) vs. rtPA patients (60%). rtPA is strongly correlated with an increased risk of ICH, especially in patients treated between 5 and 6 h after onset.
IST-3 [15] (*n* = 3035)	Time window: 0–6 h Endpoints: Primary outcome was the proportion of patients who were alive and independent (defined by an Oxford Handicap Score (OHS) of 0–2) at 6 months. Symptomatic ICH recorded at 7 days, and at 6 months. Results: Higher proportion of symptomatic ICH at 7 days in the rtPA group (7%) vs. control group (1%), and deaths at 7 days in rtPA (11%) vs. control group (7%). A similar number of fatalities in either rtPA or control groups (27% each). Despite early hazards, IV alteplase improved functional outcome.

*IV* Intravenous; *rtPA* Recombinant tissue plasminogen activator; *NINDS* National Institute of Neurological Disorders and Stroke; *mRS* Modified Rankin Score; *BI* Barthel Index; *SITS-MOST* Safe Implementation of Thrombolysis in Stroke-Monitoring Study; *NIHSS* National Institute of Health Stroke Scale; *ICH* Intracerebral haemorrhage; *TESPI* Thrombolysis in Elderly Stroke Patients in Italy; *SITS-NEW* Safe Implementation of Thrombolysis in Stroke-Non-European Union World; *ECASS* European Cooperative Acute Stroke Study; *ATLANTIS* The Alteplase ThromboLysis for Acute Noninterventional Therapy in Ischemic Stroke; *CASES* Canadian Alteplase for Stroke Effectiveness Study; *IST-3* third international stroke trial

The field of acute ischemic treatment using thrombolytic therapy before 1995 was despairing given the high rates of intracerebral haemorrhage in early clinical trials, which changed dramatically after the publication of results of NINDS stroke trial, showing benefit for IV Alteplase within 3 h of symptom onset [4]. The trial recruited 624 patients who were randomly assigned to treatment with IV Alteplase (0.9 mg/kg up to 90 mg; 10% as a bolus followed by a 60-min infusion [12]) or placebo. A significantly greater proportion of patients (38 (Alteplase) vs. 21% (placebo)) who received Alteplase showed a favourable clinical recovery at 90 days after stroke. However, the Alteplase group showed a dramatic 10-fold increase in symptomatic intracerebral haemorrhage. There was no significant difference in 90-day mortality between the two groups. Notably, less than 1% of patients experienced severe systemic bleeding at 3 months. A one-year follow-up study showed patients with AIS treated with Alteplase within 3 h after the onset of stroke were more likely to have minimal or no disability in comparison to patients receiving placebo [5, 13].

Subsequent serial observational registries and prospective randomised controlled studies conducted to investigate the safety and efficacy of IV Alteplase within the eligible time window showed a time-dependent relationship, with earlier treatment associated with larger proportional therapeutic benefits [3, 14, 15]. The ECASS-III trial showed clear benefit for patients treated between 3 and 4.5 h after stroke onset [16], which the previous RCTs failed to show because the earlier trials recruited small numbers of patients in this time window, and had treatment time windows of up to 6 h [17–19]. Other studies including the Canadian Alteplase for Stroke Effectiveness Study (CASES) registry [20], and Safe Implementation of Thrombolysis in Stroke-International Stroke Thrombolysis Register (SITS-ISTR) [21] provided further evidence of benefit for the administration of IV-rtPA therapy in the 3–4.5 h treatment window. These registries, CASES and SITS-ISTR, found comparable rates of mortality, functional independence, and incidence of symptomatic ICH within 24 h [22]. The extension of the time-window up to 4.5 h obviously offers an opportunity for treatment for more patients [21]. With regard to the treatment beyond 4.5 and up to 6 h, three trials including the Alteplase Thrombolysis for Acute Noninterventional Therapy in Ischemic Stroke (ATLANTIS-A for up to 6 h [19], and ATLANTIS-B for 3 to 5 h [18]), ECASS-II (for 0–6 h) [17], and the third International Stroke Thrombolysis Trial (IST-3) (for 0–6 h) [15] failed to demonstrate a treatment benefit for rtPA. However, a recent meta-analysis of 12 trials including 7012 patients, who received Alteplase within 6 h of onset of AIS, found significant improvement in the favourable outcome (mRS 0–1), functional independence (mRS 0–2), and survival rates at the end of final follow-up [23]. On the one hand, this analysis reinforces the need to treat patients as early as possible, though, it also suggested that some patients might benefit from Alteplase up to 6 h after stroke [15]. However, subgroup analysis for the treatment window between 4.5 and 6 h was not presented. Moreover, no significant trend toward a favourable outcome was found in a subset of patients (*n* = 4971) who received Alteplase between 3 and 6 h. The overall trend towards a beneficial outcome in 0–6-h window may have been influenced by the dominant trend observed in 0–3-h window. Pooled analysis of three trials, published in 2004, including the NINDS trials (3-h window), ECASS trials (6-h window), and two ATLANTIS trials (6-h and 5-h window), demonstrated strong association between rapid treatment and favourable outcome for patients receiving IV thrombolytic therapy within a 3 h time window [2]. Moreover, the results of two other recent pooled analyses published in 2010 (*n* = 2775 pooled from ECASS, ATLANTIS, NINDS, and Echoplanar Imaging Thrombolysis Evaluation Trial (EPITHET) trials and 2014 (*n* = 6756 pooled from IST-3, ECASS, EPITHET, ATLANTIS, NINDS) also indicated modest, yet clinically relevant, benefits to a select group in the therapeutic window of three to 4.5 h [2, 3, 14, 24]. However, risk outweighed benefit beyond 4.5 h [3, 14, 15]. In light of the emerging evidence and to provide more patients with an opportunity to receive tPA, acute stroke guidelines for the administration of rtPA following AIS have been revised by both the European Stroke Organization (ESO) [25] and American Heart Association/American Stroke Association (AHA/ASA) [26] by expanding the window of treatment from 3 h to 4.5 h. Interestingly, the Federal Drugs Agency (FDA) has not yet approved this extended indication.

Some contraindications limit the use of IV-rtPA in AIS (see Table 2) [27]. Moreover, the narrow time window of 4.5 h along with the multitude of contraindications prevent many patients from receiving treatment; indeed, less than 3% of patients presenting with AIS receive IV-rtPA [28]. Other limitations of IV-rtPA are: (a) increased rate of mortality and intracranial bleeding in internal carotid artery occlusion (ICA) [29–32] or other disabling strokes such as those with no detectable residual flow signals [33–35], (b) low recanalization rate ranging from 13 to 50% in large artery occlusion such as the proximal middle cerebral artery (MCA), the ICA, or the basilar artery [27, 35–41], and (c) unresponsiveness to large thrombi (especially when the thrombus length exceeds 8 mm [42–45], or location is proximal, such as terminal carotid artery occlusion [34, 46]. One study found only 10 and 25% of ICA and proximal MCA occlusions are recanalizable by IV-rtPA [47]. Incomplete recanalization is often observed in patients treated with IV-rtPA. For instance, 70% of patients who received IV-rtPA were found to have angiographically confirmed residual thrombus requiring complimentary intra-arterial treatment such as clot angioplasty [48].

**Table 2:** Contraindications for intravenous recombinant tissue plasminogen activator (rtPA) in acute ischemic stroke

Contraindications applicable to use of intravenous rtPA in acute ischemic stroke (AIS)
Onset of stroke symptoms more than 4.5 h.^Ψ^
History of stroke or significant head trauma in previous 3 months
Previous intracranial haemorrhage.
Symptoms are suggestive of subarachnoid haemorrhage.
Prolonged blood pressure elevation (systolic ≥185 mmHg or diastolic ≥110 mmHg).
Hypoglycemia (serum glucose <50 mg/dL (<2.8 mmol/L)).
Active internal bleeding, acute bleeding diathesis, including platelet count <100, 000/mm^3^, current anticoagulant use with an INR > 1.7, or PT > 15 s.
Heparin use within 48 h with an abnormally elevated aPTT.
Arterial puncture at noncompressible site in previous 7 days.
History of gastrointestinal tract haemorrhage within 21 days.
The recent history of major surgery intracranial or intraspinal surgery within 14 days.
Previous history of a previous aneurysm, arteriovenous malformation, or intracranial neoplasm.
Current use of a direct thrombin inhibitor or direct factor Xa inhibitors with an evidence of anticoagulation effect by laboratory tests such as aPTT, INR < ECT, TT, or relevant factor Xa activity assays.
Early ischemic changes are visible on CT in more than one-third of MCA artery vascular territory consistent with irreversible injury or evidence of haemorrhage on CT scan.

^Ψ^Additional criteria applicable for IV-rtPA between 3 to 4.5 h: patient older than 80 years, severe stroke (baseline NIHSS >25), no prior history of diabetes mellitus and AIS (both), and not currently on any oral anticoagulants regardless of INR.

*CT* Computed tomography; *INR* International normalised ratio; *IV-rtPA* Intravenous recombinant tissue plasminogen activator; *MCA* Middle cerebral artery; *NIHSS* National Institute of Health Stroke Scale; *PT* Prothrombin time; *aPTT* Activated partial thromboplastin time; *ECT* Ecarin clotting time

Novel therapies are being currently investigated to overcome the limitations of IV thrombolysis or to extend the time window of treatment, for example: (i) use of alternative fibrinolytic agents such as desmoteplase [49–53], argatroban [54], tenecteplase [19, 55], albumin [56], and plasmin [57], (ii) mixed approaches that involve combination of rtPA and other agents or therapies such as GP IIb/IIIa antagonists [58–60], antiplatelet agents (e.g., acetylsalicylic acid [61–63]), low-molecular-weight heparin [64, 65], and sonothrombolysis [66–69] to enhance microcirculatory flow, reduce residual thrombus, and boost lytic efficacy, (iii) use of non-invasive or minimally invasive methods such ventilator support [70], and pterygopalatine ganglion and petrosal nerve stimulation [71] to augment cerebral blood flow by cerebral vasodilation and alleviate blood flow steal, and (iv) endovascular procedures such as intra-arterial thrombolysis, stenting, and angioplasty to achieve greater clot manipulation and significantly higher rates of arterial recanalization [46, 72].

### Endovascular treatment

#### Intra-arterial thrombolysis

Intra-arterial (IA) thrombolysis has emerged as a promising intervention, especially for AIS patients with contraindications for IV-tPA [73–75]. Intra-arterial procedures are performed under direct visualisation; therefore, one can limit the dose of the fibrinolytic agent, mechanically manipulate the clot if required and deliver higher concentrations of the agent to the clot target (local delivery) with reduced systemic effects [76, 77]. IA can also deliver higher recanalization rates. However, IA therapy also has its unique set of challenges and disadvantages. Mechanical manipulation of a clot during IA may also increase the risk of injuring adjacent blood vessels. Moreover, advanced training is required for neuro interventionists or neuroradiologists to gain expertise in IA procedures which can be demanding [74, 78–80]. As such, endovascular treatment is only available in a limited number of specialised stroke centres.

Prolyse in Acute Cerebral Thromboembolism (PROACT) was the first randomized trial (phase II study; *n* = 46) of 6 mg recombinant pro-urokinase (rpro-UK) versus placebo undertaken in patients with angiographically documented proximal MCA occlusion to test the safety, recanalization frequency, and clinical efficacy of intra-arterial local infusion of plasminogen activators in AIS patients with symptomatic MCA occlusion of less than 6 h’ duration [81]. Investigators found a significant association of intra-arterial local rpro-UK infusion with greater frequency of recanalization in acute stroke patients with M1 or M2 occlusions compared with placebo (57.7% vs. 14.3%, *P* = 0.017). However, an increased, though not significant, symptomatic haemorrhage rate was also reported. Subsequently, a phase III, PROACT II study was undertaken, involving fifty-four stroke centers in the United States and Canada, with an increased 9 mg dosage of r-pro-UK administered over 2 h infusion while using heparin in low dose (from PROACT I) to improve the recanalization in addition to containing symptomatic brain haemorrhages [82]. Out of 180 randomied patients with AIS, treatment with IA r-pro UK within 6 h of the onset of AIS caused by MCA occlusion was significantly associated with improved clinical outcome at 90 days. The IA r-pro-UK group demonstrated significantly higher recanalization rates (66% vs. 18%, *P* < 0.001), and increased favourable independent outcome (60% vs. 18%, *P* < 0.001) than the IA heparin alone. However, higher frequency of early symptomatic intra-cerebral haemorrhage (ICH) was also observed in the intervention group (10% vs. 2%). Overall, the proportion of ICH was relatively higher in PROACT II than previous IV thrombolysis trials [81], perhaps due to greater baseline National Institutes of Health Stroke Scale (NIHSS) in PROACT II in comparison to other trials. The median baseline stroke severity in PROACT was 17, in contrast to 14 and 11 in the NINDS and ECASS II trials respectively. Contrary to the positive findings of PROACT II, another large open-label trial, Local Versus Systemic Thrombolysis for Acute Ischemic Stroke (SYNTHESIS) Expansion, found that IA thrombolytic therapy was not superior to standard treatment with IV-rtPA [83]. Disability-free outcome at 90 days was not significantly different between the IA thrombolytic and IV-rtPA groups (30.4% vs. 34.8%; adjusted odds ratio = 0.71, 95% CI 0.44–1.14). Another randomized trial, the Middle Cerebral Artery Embolism Local Fibrinolytic Intervention Trial (MELT), conducted in Japan, to investigate the safety and clinical efficacy of intra-arterial urokinase (UK) in patients within 6 h of onset of stroke with angiographically documented M1 or M2 occlusion, found a trend towards favourable outcome (defined by Modified Rankin Score (mRS) 0–2) at 90 days, and a substantial increase in likelihood of excellent outcome (defined by mRS 0–1) [84]. However, the primary endpoint (good outcome; mRS 0–2) did not reach statistical significance as the trial was aborted prematurely. A meta-analysis of five RCTs with 395 AIS patients with MCA occlusion compared IA thrombolysis with control (IV heparin) [85]. The study concluded that IA thrombolysis was significantly associated with substantial increases in recanalization rates and good (odds ratio = 2.05; 95% CI, 1.33 to 3.14; *P* = 0.001) and excellent outcomes (odds ratio = 2.14; 95% CI, 1.31 to 3.51; *P* = 0.003) in AIS. Intra-arterial thrombolytic treatment is gaining traction at some comprehensive stroke facilities at tertiary hospitals. It is often administered as an off-label therapy within 6 h of onset of stroke in patients with anterior circulation and up to 12–24 h after onset in the posterior circulation [74]. As per the guidelines of the AHA issued in 2005, and again in 2013, IA thrombolysis has been recommended in appropriately selected AIS patients with MCA occlusions within 6 h provided they were not candidates for IV-rtPA (Class I, Level of Evidence: B) [86]. The FDA has not approved IA-pro-UK.

### Mechanical Thrombectomy (MT)

MT involves a minimally invasive surgical procedure using a microcatheter and other thrombectomy devices to trap and remove the blood clot from an occluded artery. MT, delivered as a stand-alone treatment or in conjunction with systemic thrombolysis (IV-rtPA or IA thrombolysis), is currently the standard of care for AIS therapy [87]. MT devices can be classified into different subtypes based on their mechanism of action: (a) coil retriever, (b) aspiration, (c) stent-retriever, and (d) mechanical clot disruption, using laser or ultrasound. A comprehensive list of past and current MT devices is given in Table 3. Coil retriever devices and the early Penumbra aspiration system [88, 89] were the first generation of MT devices; they failed to show long-term improvements in clinical outcomes despite satisfactory revascularization efficacy (up to 50%) [90]. Coil retrievers such as MERCI [91–94], Phenox [95, 96] and Catch retrievers [97–99] use microcatheter to deliver a coiled wire across the targeted clot in the occluded artery [91–94]. Once the coil is deployed, the neuro interventionist pulls both the coil and the clot towards the catheter leading to the removal of the clot [100]. Aspiration devices such as Penumbra (Penumbra Inc., US) use vacuum aspiration to remove target clot in the occluded artery. The early generation of aspiration devices was often subject to clogging of the aspiration tips; this has been overcome in the later models by the addition of a separator wire with a bulbous tip inside the bore, which can be pushed in and pulled out by the neurointerventionist. The continual back and forth motion cleave the clot detaching it from the lumen. Eventually, the clot is sucked in, without clogging the tip, ahead of the catheter [101]. The second generation of MT devices used self-expanding stents to trap the clot by deploying them in the occluded artery [101]. They were originally conceived for stent-assisted coiling and retraction of aberrant coils dislodged during the endovascular procedures. These self-expanding stents are first lodged across the thrombus within the vessel wall, following which, once the clot is entrapped, the stent-clot combination is subsequently retracted back under constant aspiration into the delivery guide microcatheter. Some clinical trials using the new generation stent retrievers, such as Solitaire, Trevo Pro, and ReVive, have yielded recanalization rates as high as 85% in AIS with large vessel occlusion [93, 100–102].

**Table 3:** List of mechanical thrombectomy (MT) devices

MT device	Vendor	Mechanism of action	References
Merci Clot Retriever	Concentric Medical	Coil retriever	[91–94]
Phenox	Phenox, Bochum, Germany	Coil retriever/Aspiration	[95, 96]
Catch	Balt, Montmorency, France	Coil retriever	[97–99]
Distal Access Catheter (DAC)	DAC; Concentric Medical, US	Coil retriever	[129]
Early Penumbra	Penumbra Inc., US	Aspiration	[88, 89, 235–237]
AngioJet	Possis Medical, MN, USA	Aspiration/Rheolytic thrombectomy^a^	[238–241]
EKOS Primo	EKOS, Bothell, WA	Ultrasound-based on mechanical clot disruption	[242]
Neuroform	Stryker Neurovascular, US	Stent Retriever	[243, 244]
Enterprise	Codman, Raynham, MA, US	Stent Retriever	[245–247]
Solitaire	Covidien/Medtronic, Dublin, Ireland	Stent retriever	[126, 223, 248–254]
Trevo	Stryker, Kalamazoo, Michigan, US	Stent Retriever	[92, 255–259]
ReVive™	Micrus Endovascular, CA, US	Stent Retriever	[260, 261]
APERIO	Acandis, Pfzorheim, Germany	Stent Retriever	[261–263]
Embotrap Revascularization system	Neuravi, Ireland	Stent Retriever	[264]
pREset	Phenox, Bochum, Germany	Stent Retriever	[265]
The Mindframe Capture LP	Medtronic, Minneapolis, Minnesota, USA	Stent Retriever	[266, 267]
ERIC	MicroVention, CA, US	Stent Retriever	[268]
SOFIA	MicroVention, CA, US	Stent Retriever	[269–271]
Penumbra 5MAX ACE	Penumbra Inc., California, US	Aspiration	[103, 104, 106, 107, 272]
Penumbra ACE 64	Penumbra Inc., California, US	Aspiration	[105, 107, 273, 274]
Penumbra 3D separator	Penumbra Inc., California, US	Aspiration	[108, 109]
LaTIS Neurolaser laser	Latis Inc., Minneapolis, Minn	Laser recanalization based mechanical clot disruption	[275]
EPAR laser	EndoVasix, Belmont, CA	Laser recanalization based mechanical clot disruption	[130]
MicroLysUS catheter	EKOS, Bothell, WA, US	Ultrasound-based mechanical clot disruption	[276]
Wingspan	Stryker Neurovascular, Fremont, CA, US	Ultrasound-based mechanical clot disruption	[277–284]

*ERIC* Embolus Retriever with Interlinked Cage; *SOFIA* Soft Torqueable Catheter Optimized For Intracranial Access

^a^Rheolytic thrombectomy refers to the mechanical procedure of removing thrombus using multiple high-velocity, high-pressure saline jets of saline from the tip of a catheter using an AngioJet system [285]

Combined interventions, using both suction embolectomy with large bore catheters and mechanical retrieval using stent retrievers, have shown promise in recent studies [103]. In this technique, aspiration of the clot, a cheaper alternative, is attempted first using a large bore microcatheter such as the Penumbra MAX systems [104–107]. If the aspiration fails, mechanical retrieval is attempted by inserting the stent retrievers via the aspiration catheter. Using this sequential combination, phenomenal recanalization rates of up to 95% have been achieved [103], compared with stand-alone direct aspiration rates of 78%. Another application of combined approach is the latest generation Penumbra device called Penumbra 3D separator, which incorporates lesional aspiration technology coupled with an advanced stent retriever device, allowing the breakdown of a clot in addition to radial direction using stent struts [100, 108]. Penumbra 3D separators have demonstrated good revascularization of large vessel occlusions and a greater rate of functional independence at 90 days [108, 109].

A detailed comparative overview of successful clinical trials concerning endovascular treatment for AIS along with the imaging and clinical selection criteria, and the outcome measures are presented in Table 4.

**Table 4:** Comparison of baseline characteristics and outcome measures of the recent endovascular trials [27, 194, 286]

Trials	MR CLEAN [110]	ESCAPE [111]	EXTEND-IA [76]	SWIFT PRIME [112]	REVASCAT [113]	THERAPY [128]	THRACE [127]
Region	Netherlands	United States, Canada, South Korea, Ireland, United Kingdom	Australia and New Zealand	United States and Europe	Spain	United States	France
Number of centres	16	22	10	39	4	4	26
Number of patients; n (CG/IA)	500 (267/233)	315 (150/165)	70 (35/35)	196 (98/98)	206 (103/103)	108 (54/54)	412 (208/204)
BASELINE CHARACTERISTICS							
Age Range	≥ 18	≥ 18	≥ 18	18–80 years	18–80 years	18–85	18–80
NIHSS Range	≥ 2	> 5	N.R.	8–29	≥ 6	≥8	10–25
Control group	Standard medical therapy (+/− IV tPA)	Standard medical therapy (+/− IV tPA)	IV-tPA only	IV-tPA only	Standard medical therapy (+/− IV tPA)	IV-tPA only	IV-tPA only
Intervention group	IAT	IAT	ET with Solitaire FR stentriever	ET with Solitaire FR stentriever	ET with Solitaire FR stentriever	ET with Penumbra aspiration system	Endovascular MT
Intervention using Stent retriever in IA arm	81.5%	86.1%	100%	100%	100%	0%	N.R.
Time window	0–6 h	0–12 h	0–6 h	0–6 h	0–8 h	0–4.5 h	0–5 h
Neurologic inclusion criteria	N.A.	Barthel Index of ≥90	mRS scores of 0–2	mRS scores of 0–1	mRS scores of 0–1		
Neuroimaging techniques	CT/CTA	CT/CTA/CTA Multiphase (for collaterals)	CT/CTA/CTP (for mismatch)	CT/CTA/MRA/MRP/CTP (for infarct core)	CT/CTA(MRA/DSA)	CT/CTA	CT/CTA
Large artery occlusion	CTA	CTA	CTA or MRA	CTA or MRA	CTA or MRA	CTA	CTA or MRA
Affected arteries	TICA, M1, M2, A1, A2	TICA, M1	TICA, M1, M2	TICA, M1, M2	TICA, M1	MCA	ICA, M1, TB, M2
Infarct core/perfusion	N.R.	NCCT, CBV or CBF ASPECTS ≥6	Core^Ψ^ < 70 ml (>1.2)^¥^	CoreΦ< 50 ml (>1.8)^¥^ NCCT ASPECTS ≥6	NCCT ASPECTS ≥7 DWI ASPECTS ≥6	Clot length ≥ 8 mm	N.R.
Collateral status	N.R.	Good/Moderate	N.R.	N.R.	N.R.	N.R.	N.R.
Median stroke onset to groin puncture	260 min	241 min	210 min	224 min	269 min	226 min	255 min
Baseline NIHSS [Median (IQR)]; CG vs IA	18 (14–22) vs17 (14–21)	17 (12–20) vs 16 (13–20)	13 (9–19) vs 17 (13–20)	17 (13–19) vs 17 (13–20)	17 (12–19) vs 17 (14–20)	N.R.	17 (13–20) vs 18 (15–21)
Median ASPECTS (%); CG/IA	9/9	9/9	NR/NR	9/9	8/7	N.R.	N.R.
Patients Receiving IV-rtPA (%); CG/IA	91/87	79/73	100/100	100/100	78/68	100/100	100/100
STUDY OUTCOMES							
Primary Outcomes	Shift in mRS at 90 days	Shift in mRS at 90 days	Reduction in perfusion lesion volume; NIHSS reduction ≥8 pointsor mRS score of 0–1 at day 3	Distribution of mRS at 90 days; % mRS 0–2 at 90 days	Shift in mRS at 90 days	Shift in mRS at 90 days	Shift in mRS at 90 days
mRS (0–2) at 90 days %; CG vs IA	19.1 vs 32.6, *P* < 0.05	29.3 vs 53, *P* < 0.001	40 vs 70, *P* = 0.001	35.5 vs 60.2, *P* < 0.001	28.2 vs 43.7	30.4 vs 38	42.1 vs 54.2
Improvement in mRS 0–2 at 90 days	13.5%	23.7%	31.4%	24.7%	15.5%	7.6%	12.1%
sICH risk (%); CG vs IA, P	6.4 vs 7.7, *P* > 0.05	2.7 vs 3.6, *P* > 0.05	5.7 vs 0, *P* > 0.05	3.1 vs 0, *P* = 0.12	1.9 vs 1.9, *P* > 0.05	11.3 vs 10.9	2 vs 2, *P* = 0.71.
Parenchymal Hematoma Risk (%); CG/IA	6 vs 6	2.0 vs 4.8	8.6 vs 11.4	N.R. vs N.R.	5.8 vs 5.8	N.A.	9.45 vs 13.8, *P* = 0.53
Mortality (%); CG vs IA, P	22.1 vs 21, *P* > 0.05	19 vs10.4, *P* = 0.04	20 vs 8.6, *P* > 0.05	12.4 vs 9.2, *P* > 0.05	15.5 vs 18.4, *P* = 0.06	23.9 vs 12	13 vs 12; *P* = 0.70
Decrease in mortality at 90 days	1.1%	8.6%	11.4%	3.2%	−2.9%	11.9%	1%
Complete recanalization rates							
*mTICI Score 2b/3*	58.7%	72.4%	86.2%	88.0%	65.7%	N.R.	N.R.
*Complete recanalization based on neuroimaging 24–27 h later; CG* vs *IA*	68/207 (33%)^a^ vs 141/187 (75%)^a^	N.A. vs N.A.	15/35 (43%)^a^ vs 33/35 (94%)^a^	21/52 (40%)^b^ vs 53/64 (83%)^b^	N.A. vs N.A.	N.A.	N.A.
Brain infarction volume at 24 hc (mean, 95% CI); CG vs IA, P	79 mL (34–125) vs 49 mL (22–96), *P* < 0.01	N.A. vs N.A.	N.A. vs N.A.	35 mL (0–407) vs 32 mL (0–503), *P* = 0.09	39 mL (12–87) vs 16 mL (8–59), *P* = 0.02	N.A.	N.A.
NNT	7.1	4.2	3.2	4.0	6.3	13.2	8.3

*IQR* Interquartile range; *TICA* Terminal internal carotid artery (Carotid T/L); *M1* and *M2* Branches of the MCA; *A1* and *A2* Branches of the ACA; *mTICI* Modified Thrombolysis in Cerebral Infarction; *mRS* Modified Rankin Scale; *N.R*. Not required; *N.S*. Not significant; *N.A*. Not available; *CTA* Computed tomography angiography; *NCCT* Non-contract computed tomography; *CBV* Cerebral blood volume; *CBF* Cerebral blood flow; *MRA* Magnetic resonance angiography; *ASPECTS* Alberta Stroke Program Early CT score; *CG* Control group; *IA* Intervention arm; *IAT* Intra-arterial therapy; *TB* Upper third of the basilar artery; *MCA* Middle cerebral artery; *M2* Insular portion of the MCA; *M1* Proximal portion of the MCA; *ET* Endovascular Thrombectomy; *MT* Mechanical thrombectomy; *ESCAPE* Endovascular Treatment for Small Core and Anterior Circulation Proximal Occlusion With Emphasis on Minimizing CT to Recanalization Times; *EXTEND-IA* Extending the Time for Thrombolysis in Emergency Neurological Deficits—Intra-Arterial; *MR CLEAN* Multicentre Randomized Clinical Trial of Endovascular Treatment for Acute Ischemic Stroke in the Netherlands; *NIHSS* National Institutes of Health Stroke Scale; *REVASCAT* Randomized Trial of Revascularization with Solitaire FR Device Versus Best Medical Therapy in the Treatment of Acute Stroke Due to Anterior Circulation LVO Presenting within 8 h of Symptom Onset; *SWIFT PRIME* Solitaire with the Intention for Thrombectomy as Primary Endovascular Treatment; *NNT* Number needed to treat for benefit (mRS score 0–2); *THRACE* Mechanical thrombectomy after intravenous alteplase versus alteplase alone after stroke

^¥^Target mismatch ratio; ^λ^ Sum of median of parameters; ^Φ^The ischemic core was assessed by MRI or CT; ^Ψ^The ischemic core was defined by regional cerebral blood flow on CT perfusion or diffusion-weighted imaging; ^a^Recanalization shown in brain CTA/MRA at 24 h; ^b^Reperfusion shown in brain CT perfusion/MR perfusion at 27 h; ^c^Brain infarction volume at 24 h after treatment measured with CT in MR CLEAN trial and with CT or MRI in SWIFT PRIME and REVASCAT trials

Following the success of five multicentre, open-label, randomized controlled endovascular acute stroke trials including MR CLEAN (Multicenter Randomized Clinical Trial of Endovascular Treatment for Acute Ischemic Stroke in the Netherlands) [110], ESCAPE (Endovascular Treatment for Small Core and Anterior Circulation Proximal Occlusion with Emphasis on Minimizing CT to Recanalization Times) [111], SWIFT PRIME (Solitaire With the Intention for Thrombectomy as Primary Endovascular Treatment of Acute Ischemic Stroke) [112], EXTEND-IA (Extending the Time for Thrombolysis in Emergency Neurological Deficits–Intra-arterial) [76], and REVASCAT (Randomized Trial of Revascularization with Solitaire FR Device vs Best Medical Therapy in the Treatment of Acute Stroke Due to Anterior Circulation Large Vessel Occlusion Presenting within 8 h of Symptom Onset) [113], it is now accepted that the combined treatment with second-generation stent retriever MT devices and IV-rtPA within 6 h after stroke onset is superior to standard medical therapy (with IV-rtPA alone) for AIS caused by a proximal large artery occlusion of the anterior circulation [114].

Immediately after the announcement of the MR CLEAN results [110], the other four trials were terminated prematurely for interim analysis. The results of these trials were then published in quick succession in late 2014 and early 2015. The number needed to treat (NNT) for these five trials ranged from a minimum of three (EXTEND-IA) to a maximum of 7.4 (MR CLEAN). The success of these trials has revolutionised stroke therapy. However, given that only trained neuro-interventionists can perform the MT procedures, stroke care facilities should expeditiously work on integrating MT with standard care, to minimise the time required for imaging and preoperative preparation needed for this therapy [115]. A recent meta-analysis, based on a pooled analysis of 1287 patients, published by the Highly Effective Reperfusion evaluated in Multiple Endovascular Stroke Trials (HERMES) collaboration, showed that endovascular MT added to the best medical therapy more than doubles the odds of a higher rate of (a) functional independence (mRS score at 90 days of 0 to 2) (46% vs. 27%, odds ratio 2.35, 95% CI 1.85–2.98) [116], and (b) significantly reduced disability (improvement of ≥1 points on the mRS at 90 days) (adjusted OR 2.49, 95% CI 1.76–3.53) compared with best medical therapy alone in AIS patients with large vessel occlusion in the anterior circulation. The rates of symptomatic ICH or 90-day mortality were also not significantly different between the endovascular MT and control groups [117]. Another recent meta-analysis by the same HERMES collaboration has suggested that the endovascular MT plus medical therapy is beneficial up to 7.3 h after the onset of stroke [118]. Recently published DAWN trial has shown benefit of MT for an extended time window of 6–24 h [119]. To sum up, these studies make a convincing case for the administration of early thrombectomy using second-generation stent retrievers for limiting post-stroke disability in patients with large vessel occlusion in the anterior circulation. They also reinforce the importance of early treatment based on an inverse association between time to endovascular reperfusion and better functional outcome [118].

#### Clinical trials with first-generation devices

Prior to MR CLEAN trial, three endovascular trials, using mainly first generation MT devices during 2004 and 2012, including MR RESCUE (Mechanical Retrieval and Recanalization of Stroke Clots Using Embolectomy) [120], the IMS (Interventional Management of Stroke) III [121], and the SYNTHESIS Expansion (Intra-arterial vs. Systemic Thrombolysis for Acute Ischemic Stroke) [83] failed to demonstrate any functional benefit for intra-arterial treatment in AIS. The results of these trials, which were published in 2013, raised concerns regarding the efficacy of endovascular MT in large vessel occlusion. Potential causes for this lack of effect were suggested including: (a) the three trials used old-generation devices with reduced recanalization efficacy in comparison to the later generation devices which were used in the MR CLEAN trial, and (b) previous trials did not use vessel occlusion as an eligibility criterion because radiological confirmation of large artery occlusion was lacking due to limited availability of CTA at that time [122–124].

#### Phase 2 clinical trials with second-generation devices

The second-generation MT devices, Solitaire flow restoration device and Trevo retriever, are based on stent retrievers, which are very effective in capturing thrombus and produce excellent vessel recanalization. Two phase 2 clinical trials, SWIFT (Solitaire Flow Restoration Device Versus the Merci Retriever in Patients with Acute Ischemic Stroke) and TREVO 2 (Trevo Versus Merci Retrievers for Thrombectomy Revascularization of large vessel occlusion in Acute Ischemic Stroke), demonstrated that stent retrievers achieved better clinical outcomes by increasing recanalization of large artery occlusions, than the first-generation Merci Retriever and Penumbra System devices [93, 94, 125, 126]. The SWIFT study assigned eligible patients to receive MT treatment with either MERCI device (*n* = 55) or Solitaire stent retriever (*n* = 58). The Solitaire group showed significantly improved recanalization, as defined by a Thrombolysis in Myocardial Infarction (TIMI) Score of two (partial recanalization) or three (total recanalization) (61% vs. 24%, *P* < 0.0001), and better functional outcomes (58% vs. 33%, *P* < 0.0001) than the MERCI group. The TREVO trial produced similar outcomes, with the TREVO device achieving better recanalization (86% vs. 60%, *P* < 0.0001), defined by Thrombolysis in Cerebral Infarction (TICI) score of 2 or 3, and improved long-term clinical outcomes (40% vs. 22%, *P* < 0.013) in comparison to the first generation Merci retriever. No difference in the incidence of symptomatic intracerebral haemorrhage (sICH) was reported between TREVO and Merci groups.

#### Phase 3 clinical trials with second-generation devices

MR CLEAN was a Dutch endovascular trial that assigned 500 patients to the intervention arm (IAT with standard therapy; *n* = 233) and the control arm (standard therapy; *n* = 267) alone [110]. All patients in the control arm received IV-rtPA. Endovascular procedures were performed using second-generation stent retrievers. Stent retrievers were used in 81.5% (190/233) of patients who received IA treatment. MR CLEAN included patients presenting within 6 h of stroke onset, aged 18 and above, with a minimum NIHSS score of two, and large artery occlusion confirmed on CTA. MR CLEAN trial did not have an upper age threshold for eligibility unlike previous trials, where patients above 80 years were excluded. Moreover, an Alberta Stroke Program Early CT Score (ASPECTS) score was not used in the randomization of patients. Patients who received MT and standard treatment (intervention arm) showed good recanalization (defined by TICI score of 2b (>50% recanalization) or 3) in 59% (115/196) of the patients. This arm also demonstrated improvement in good outcome at 90 days (32.6% vs. 19.1%; OR = 1.67 [95% CI 1.21 to 2.3]), and a significant decrease in brain infarct at 24 h (49 mL vs. 79 mL, *P* < 0.01) in comparison to the standard medical therapy group. The number need to treat (NNT) was 7.1. No significant difference in the occurrence of sICH or death was found in between the two groups.

The Endovascular Treatment for Small Core and Proximal Occlusion Ischemic Stroke (ESCAPE) trial randomised 315 patients, 150 in the control arm and 160 in the MT arm, for an extended therapeutic time window of 0–12 h [111]. Strict inclusion criteria including NIHSS > 5, pre-morbid Barthel index of 90 and above, large artery occlusion confirmed on CT angiography (CTA), ASPECTS score of ≥6, and good or moderate leptomeningeal collateral status on CTA was applied. In comparison to previous trials, ESCAPE used CTA, preferably multiphase CTA, to select patients based on the neuroimaging assessment of the site of occlusion and collateral status. The trial was unique given the pioneering enrollment goals, including door to puncture times of less than 60 min, and door to recanalization time of fewer than 90 min. Given the trial was stopped following the announcement of MR CLEAN results, an interim analysis showed good recanalization rates (72%), defined by TICI score of 2b or 3, and significantly better functional outcomes in the MT group vs. the standard therapy (53% vs. 29%, *P* < 0.001). Concerning safety endpoints, MT showed reduced mortality rate (10% vs. 19%, *P* = 0.04), and comparable sICH rates (3.6% vs. 2.7%, *P* > 0.05) vs. the control group.

The SWIFT-PRIME trial boasted the maximum number (*n* = 39) of recruitment centres spanning the United States and Europe [112]. Between 2012 and 2014, 196 patients presenting within 6 h of stroke onset were enrolled and assigned to treatment groups: MT (using Solitaire FR stentriever) plus standard therapy, IV-rtPA (*n* = 98), or standard therapy alone (n = 98). For randomization, patients with NIHSS scores between 8 and 30, aged between 18 and 80 years, and a premorbid score of mRS < 1 were considered. The SWIFT-PRIME study used neurovascular angiography imaging (CTA/magnetic resonance angiography (MRA)) to identify patients with large artery occlusion prior to randomization. SWIFT PRIME introduced an automated post-processing imaging pipeline software for penumbra profiling (RAPID), although its use was not mandatory or part of the inclusion criteria. Infarct core volume was estimated from CT Perfusion (CTP) using RAPID. A cut-off of infarct core volume of >50 mL on CTP was used to exclude patients. The ischemic core was defined by regional cerebral blood volume (CBV) or delayed time to peak (TTP) of the residual function. Moreover, patients with baseline ASPECTS score of less than six were excluded. The SWIFT PRIME study achieved the highest recanalization (defined by modified TICI score of 2b or 3) rate of 88% for the MT arm in comparison to the other four contemporary endovascular trials. The MT group showed 24.7% improvement in the functional outcome at 90 days (mRS 0–2: MT 60.2% vs standard therapy 35.5%, *P* < 0.001). Efficacy of MT in SWIFT-PRIME (60.2%) was higher than MR CLEAN (32.6%) and ESCAPE (53%) trials. No significant group differences in sICH risk (0% vs 3%, *P* = 0.12), or 90-day mortality (9% vs 12%, *P* > 0.05) were found.

EXTEND-IA was an Australasian trial involving 10 centres across Australia and New Zealand that enrolled 70 patients who were randomised to receive IV-rtPA alone, or MT using Solitaire FR stentriever plus IV-rtPA [76]. Like the SWIFT PRIME, EXTEND-IA also required administration of MT and IV-rtPA within 6 and 4.5 h of onset of stroke symptom, respectively. However, unlike other trials, there was no restriction on stroke severity as a criterion for patient inclusion. Moreover, age or ASPECTS score was not part of criteria for randomization. Pre-morbid functional status of mRS 0 to 2 was required for inclusion in the study. With regards to neuroimaging selection criteria, CTP for detection of favourable penumbra using RAPID software similar to SWIFT PRIME (CTP core volume less than 70 mL or mismatch ratio greater than 1.2), and CTA for large artery occlusion diagnosis were used to randomise patients. The primary outcome for the EXTEND-IA study was a reduction in the NIHSS score of 8 or higher, a score of 0 or 1 on mRS at 90 days, or reduction in perfusion lesion volume. EXTEND-IA showed the highest improvement in functional outcome at 90 days in comparison to the other four-endovascular trials (31.4%), and recanalization rate (86.2%) comparable to SWIFT PRIME. The number needed to treat (NNT) was the lowest for EXTEND-IA (NNT: 3.2 (EXTEND-IA) vs. 7.1 (MR PRIME) vs. 4.2 (ESCAPE) vs. 4 (SWIFT-PRIME) vs. 6.3 (REVASCAT)). The MT arm showed significantly improved functional outcome compared to the standard therapy arm (70% vs. 40%, *P* = 0.001). The investigators reported no significant differences in risk of sICH and mortality between the two groups.

REVASCAT was a Spanish trial involving four tertiary hospitals that enrolled 206 AIS patients presenting within 8 h of the symptom onset randomised to receive MT with the Solitaire FR plus standard medical therapy (*n* = 103) or standard medical therapy including IV-rtPA for eligible patients (n = 103) [113]. Clinical inclusion criteria included age between 18 and 80 years, NIHSS of 6 or more, and a pre-treatment mRS score of 0 to 1. Neuroimaging with CTA or MRA was used to select patients with large vessel occlusion. Moreover, only patients with ASPECTS scores of seven and above on NCCT or six and above on DWI MRI were included. Interestingly, the inclusion criteria were revised to age < 85 years and ASPECTS score of eight or above on NCCT after the enrollment of 160 patients. The REVASCAT trial showed 65.7% recanalization for patients in MT arm, and a significant reduction in brain infarction volume at 24 h (16 mL vs. 39 mL, *P* = 0.02) vs. standard medical therapy. No significant differences were noted in the rates of sICH (1.9% vs. 1.9%, *P* > 0.05) or mortality (18.4 vs. 15.5, *P* = 0.06) between the two groups. Patients in the MT arm were more likely to have a better functional outcome at 90 days (43.7% vs. 28.2%; OR 2.1, 95% CI 1.1 to 4).

In summary, these five-endovascular randomised controlled trials have consistently shown that MT significantly improves reperfusion and functional outcome at 90 days without an increase in mortality compared to patients receiving standard medical therapy. Use of advanced imaging features such as ASPECTS scale or perfusion imaging may assist in selecting patients who are most likely to benefit from a combined approach. Advanced imaging helps to differentiate between salvageable tissue and irreversibly dead core. Unfortunately, selection of patients using advanced imaging excludes many patients who may otherwise have received intra-arterial treatment.

Two new clinical trials, the Trial and Cost-Effectiveness Evaluation of Intra-arterial Thrombectomy in Acute Ischemic Stroke (THRACE) [127] and the Assess the Penumbra System in the Treatment of Acute Stroke (THERAPY) [128], have addressed these shortcomings by keeping selection criteria to a minimum except for the use of angiographic technique such as CTA or MRA to localize and confirm the arterial occlusion. The THRACE trial, conducted across 26 centres in France, included 336 patients, aged 18 to 80 years and NIHSS score ranging between 10 and 25, presenting within 5 h of symptom onset with moderate to a severe stroke caused by the large artery occlusion of the anterior circulation (radiologically confirmed on CTA), out of which 195 received IVT, and 141 received combined IVT and MT treatment, without a selection based on advanced imaging-based criteria. Combined IV-rtPA and MT provided a higher rate of functional outcome at 90 days (54.2% vs. 42.1%). No significant differences in mortality and sICH risks were noted between the MT and control arms. The THERAPY trial, undertaken across four centres in the United States, selected patients with AIS presenting within 4.5 h of symptom onset who have evidence of large clot burden (clot length ≥ 8 mm) in the anterior circulation [128]. CTA was used to identify patients with large vessel occlusion. Patients with NIHSS score of eight and above and age between 18 and 85 years were included for randomization. Patients were then treated with both combined IV-rtPA and IAT with the Penumbra aspiration system (a new technique of aspiration thrombectomy) or with the IV-rtPA alone. The results showed a positive trend, though not significant, of aspiration thrombectomy towards better functional outcomes (38% vs. 30.4%). Interestingly, the intervention arm showed a considerable reduction (11.9%) in mortality (mortality in intervention arm vs. control arm: 12% vs. 23.9%). No significant difference in sICH risk was observed (10.9% vs. 11.3%).

To sum up, MT has a number of advantages over systemic thrombolysis [87]: (a) MT yields higher rates of revascularization, and reduced rates of long-term functional dependence, in a select group of AIS patients with large vessel occlusion, compared to IVT alone [78, 79, 129–131], (b) MT extends the therapeutic time-window for acute intervention up to 24 h from stroke onset, beyond the 4.5 h restricted time window for IVT, which is applicable to only a small number of AIS patients [74, 119, 132–134], (c) MT presents a viable alternative to patients with large vessel occlusion who respond poorly, vis a vis poor recanalization rates and risk of haemorrhage, with systemic therapy [134], or those who have contraindications to the use of systemic thrombolysis, and (d) MT is efficient in dissolving clots which are resistant to enzymatic degradation, such as old and large clots with hardened fibrin and cross-linked thrombi containing calcium and cholesterol crystals, which have poor recanalization yields with systemic thrombolysis [93, 133, 135].

### Combined intravenous and endovascular therapy: A multimodal reperfusion therapy (MMRT) approach

The idea behind combining intravenous and endovascular approaches, also called bridge therapy [136–141] or multimodal reperfusion therapy (MMRT), is to take advantage of best of both approaches by allowing fast and early access to IV-rtPA within the first 4.5 h of stroke onset, and superior recanalization rates even for delayed time windows beyond 4.5 h using endovascular therapy [142]. Notably, the Interventional Management of Stroke (IMS) I [143] and II [144] trials were conducted to investigate the feasibility and safety of combined interventions: low dose, 0.6 mg/kg, IV-rtPA followed by intra-arterial rtPA within 3 h since stroke onset, to recanalization of AIS. The bridge IV and IA therapy were not significantly different from the IV-rtPA alone as both yielded similar proportions of ICH, rates of mortality and mRS at 90 days. The subsequent IMS III trial also found no additional benefit of bridge therapy compared with IV-rtPA alone [145]. One of the important factors that may have played a role in these results is the time delay (approximately 32 min) between the IV-rtPA and initiation of intra-arterial therapy [121, 143]. Unlike the neutral results of IMS III trial, a recent meta-analysis reported a significantly strong association of combined intravenous-IA thrombolysis over IV fibrinolysis alone with favourable outcome, reduced mortality, and improved recanalization rates [146].

The Stent-Assisted Recanalization in Acute Ischemic Stroke (SARIS) trial, conducted to investigate the safety of intracranial stent deployment within 8 h of stroke onset, demonstrated expeditious recanalization, and favourable outcomes at 30 and 180 days clinical follow-up [147, 148]. Stent deployment averts arterial reocclusion and thrombus reformation in cases with partial embolectomy or arterial stenosis. The utility of self-expanding stents (SES) has been explored. The SES yields dramatically high recanalization rate of up to 90% by a combination of balloon angioplasty and stent implantation [147]. Undoubtedly, the bridge endovascular therapy using MT, intracranial stent deployment, and IV thrombolysis allows an extended time window, and therefore a higher proportion of revascularisations and improved clinical outcomes in AIS patients with large artery occlusion of anterior circulation [22].

A number of MMRT approaches including combined IV-rtPA and IA-tPA [136–138, 140, 141, 149], IV-tPA followed by multimodal endovascular therapy [150], combined IA thrombolytics and glycoprotein IIb/IIIa inhibitors [151], IA administration of microbubbles and continuous 2-MHz ultrasound insonation [139], IA-tPA followed by stenting [152, 153] or angioplasty or both [154, 155], IA urokinase and mechanical clot disruption following failed IV-tPA [156], MT using balloon angioplasty and adjuvant systemic thrombolysis (IV-tPA, IA urokinase, both IV-tPA and IA-urokinase, and IV and/or IA eptifibatide) [157], MT (MERCI retrieval, angioplasty/stent) with or without adjunctive IA-tPA/Urokinase [158], MT using clot retrievers and angioplasty with intracranial or extracranial stenting [151], have demonstrated considerably improved recanalization, reperfusion and clinical outcomes [140, 150, 151, 154, 155, 159, 160]. Gupta et al. (2011) reported significantly higher recanalization rates for multimodal therapy (MT using intracranial stenting in conjunction with IV/IA thrombolysis) (74% [435/584]) in comparison to pharmacologic treatment only (61% [160/264]), or MT only (63% [173/274]) in a large retrospective cohort of 1122 AIS patients involving the anterior circulation who received IAT within 8 h of stroke onset [153].

Drawing from the success of these studies, an endovascular MMRT approach using pharmacological thrombolytics (IA lytic drugs), in conjunction with MT using mechanical devices such as clot retrievers, angioplasty with stenting, aspiration devices, and stent retrievers is being increasingly adopted as the treatment of choice for stroke due to large vessel occlusion [153, 159]. Endovascular MMRT or bridge therapy offers a safe alternative for AIS patients, with large intracranial vessel occlusion, who fail to reperfuse with systemic thrombolytic drugs.

### Role of collaterals in penumbral sustenance and recanalization

Recanalization is positively associated with favourable clinical outcome and increased survival rates in acute ischemic stroke [39, 40, 161–165]. A meta-analysis of 53 studies encompassing 2066 patients reported strong association of recanalization with the good functional outcome (OR 4.43, 95% CI 3.32 to 5.91), and reduced mortality (OR 0.24, 95% CI 0.16 to 0.35) at 3 months in AIS [146]. The rate of recanalization classified based on intervention: spontaneous, IV fibrinolytic, intra-arterial fibrinolytic, combined IV and IA thrombolysis or MT was 24.1, 46.2, 63.2, 67.5, 83.6%, respectively. Early recanalization is associated with rapid clinical improvement in some patients [166]. However, despite early recanalization, some patients who are otherwise unresponsive to treatment over short-time follow-up, may show delayed recovery or favourable long-term outcome suggesting the possibility of an “ischemic stunning” or “stunned brain” syndrome [167]. Recanalization enables restoration of cerebral blood flow to the hypoperfused brain region surrounding the infarcted ‘core’. This area of the brain with reversible ischemia surrounding the infarcted core is called ischemic penumbra [168]. Animal studies have indicated that considerable salvage of penumbral tissue is possible on the restoration of blood flow to the hypoperfused brain area, even after 24 h, irrespective of time of reperfusion [169, 170]. From a pathophysiological standpoint, the survival of penumbra, independent of time, for up to 48 h has been reported using a ligand that selectively binds to hypoxic but viable tissue ([(18)F]fluoromisonidazole), and positron emission tomography (PET) on consecutive patients presenting within 48 h of AIS [171]. As expected, the penumbra reduces over time; it is observed in 90–100% of stroke patients in the first 3 h after stroke onset [172], 75–80% of patients 6 h after stroke onset, and approximately 33% of patients 18 h after stroke onset [173].

Despite the success of endovascular procedures, a number of patient-specific factors may determine the response to treatment such as (a) collateral circulation [174–176], (b) site of vessel occlusion [48], (c) onset to angiographic reperfusion time [177–179], (d) hyperglycemia [180–182], and (c) location of cerebral ischemia [174, 183]. In the case of cerebral ischemia, collaterals compensate for the sudden drop in CBF in the hypoperfused area by the maintenance of a continual blood supply to ischemic penumbra. For recanalization to translate into positive outcomes, adequate collaterals must delay the infarction of tissue until recanalization is achieved [184]. Collaterals can sustain the penumbra even in the absence of reperfusion or recanalization. According to a meta-analysis, good baseline collaterals were associated with favourable outcome at 90 days, decrease in the risk of symptomatic ICH, and decrease in risk of death at 90 days in patients with AIS receiving endovascular treatment [176]. Good baseline collaterals have been found to be associated with 24 h perilesional hyperperfusion [185], good clinical outcome [176, 186–189], lower rates of symptomatic ICH and mortality [176], improved radiologic outcome [188], cortical infarct volume [190], good reperfusion [191], and stroke severity [191]. On the other hand, delayed-cortical vein filling was independently associated with reduced baseline collateral status in AIS [185]. Collateral grading is used to determine recanalization rate after endovascular reperfusion therapy [175, 192]. Collateral pathophysiology may have predictive value, and patent collaterals may help boost reperfusion [193]. Some scales and grading tools are available for reliable and quick assessment of the patency of collaterals by visual examination of multiphase CTA [189].

### Limitations and unresolved questions of endovascular reperfusion therapy

Despite the substantial advantages of MT, this is only offered to a limited number (5–10%) of AIS patients [131]. Currently, vascular imaging such as CTA or MRA is not routinely performed at the time of presentation. The treating physician needs to make a rapid decision based on the vascular imaging (whether or not angiography shows a large vessel occlusion), assessment of ischemic penumbra (based on CT or MR perfusion imaging), time since the onset of stroke symptoms, along with other baseline clinical factors and previous history (such as significant past trauma, haemorrhage or stroke) [90, 127, 194]. The need for advanced imaging, especially CTA, implies that only a few select AIS patients would be suitable for MT. MT with current devices is not well suited for AIS with occlusions in distal locations and with penetrator occlusions due to difficulty in navigating with the catheter, increased risk of intraprocedural vessel perforation, and a higher risk of mortality [101, 195]. Also, patients with in situ intracranial atherothrombi (in situ atherosclerotic plaque with supervening thrombosis), may be more suited for balloon angioplasty and stenting over MT [101]. MT yields superior recanalization rates with occlusions of cardioembolic origin [196] or proximal aortocervical arterial source [101]. Delayed treatment may lead to further shrinking of salvageable penumbral tissue due to absence or insufficient reperfusion. MT is a highly specialized interventional procedure; as such, it is strongly recommended that MT be performed in a comprehensive stroke unit with a well-equipped neurointervention suite by an interventional neuroradiologist or an endovascular specialist with experience in the procedure, or a credentialed neurointerventionist along with a team of stroke neurologists, and nursing staff [197]. Lack of experienced practitioners (such as credentialed neuro interventionist, endovascular specialists) [197], along with logistic difficulties such as prehospital delays (e.g., prolonged transfer time to and from rural hospitals), and inter-facility transfers, make MT unlikely to be widely implemented. A comprehensive national and international stroke care policy needs to be adopted to address these logistical and other systemic deficiencies within the relevant healthcare systems.

In addition to the limitations of present reperfusion therapies in AIS settings, a number of unresolved questions or issues need to be addressed.

#### Extending the time window of MT beyond 6 h

Evidence for efficacy and safety of endovascular reperfusion beyond 6 h is still insufficient given that no randomised trial has used this as an inclusion criterion. However, a recent meta-analysis has indicated the benefit of endovascular MT plus medical therapy up to 7.3 h after the symptom onset [118]. Some trials including the premise of the Diffusion and Perfusion Imaging Evaluation for Understanding Stroke Evolution (DEFUSE 3), the DWI or CTP Assessment With Clinical Mismatch in the Triage of Wake-Up and Late Presenting Strokes Undergoing Neurointervention (DAWN), the Perfusion Imaging Selection of Ischemic Stroke Patients for Endovascular Therapy (POSITIVE) and Imaging-Guided Patient Selection for Interventional Revascularization Therapy (START) trials seek to extend the time to endovascular reperfusion. DEFUSE 3 selects AIS patients using penumbral mismatch on CTP or MRI who may benefit from MT between 6 h and 16 h post onset [198]. Similarly, the goal of the POSITIVE trial is to use appropriate imaging selection to improve stroke-related disability, and functional outcome in AIS patients treated with MT presenting between 6 to 12 h who are either ineligible for or refractory to IV-tPA treatment [199]. DAWN, which recruited patients in which treatment with Trevo MT was initiated within 6–24 h after stroke onset [200] [201], was terminated early after an interim analysis of the first 200 patients demonstrated a 73% relative risk reduction in disability (48.6% in MT group vs. 13.6% in control group). DAWN study has confirmed that advanced imaging based patient selection outweighs time-based decision making in acute ischemic stroke [119]. This has major implications for treatment in acute ischemic stroke given that DAWN showed efficacy over an extended time window from 6 to 24 h [202].

#### General anaesthesia vs. conscious sedation

The type of anaesthesia, whether general anaesthesia (GA) or conscious sedation, has implications for outcomes in AIS patients during and after endovascular treatment [203]. This has been a subject of ongoing debate given that several retrospective studies have hypothesised that GA may be associated with periprocedural hypotension that may cause poorer clinical outcome, despite the procedural advantages of GA [203–205]. A consensus is still missing over the anaesthetic management of AIS patients during IAT [206]. The MR CLEAN pre-trial study group found that non-GA was significantly associated with good clinical outcomes in AIS patients undergoing IAT [206]. Three randomized trials, Sedation vs Intubation for Endovascular Stroke Treatment (SIESTA) [207], the General or Local Anaesthesia in Intra-arterial Therapy (GOLIATH) [208], and the Sedation Versus General Anaesthesia for Endovascular Therapy in Acute Stroke (ANSTROKE) [209], aimed to investigate the impact of anaesthesia type on neurological outcome in IAT, and whether conscious sedation is the optimal anaesthesiologic management modality in endovascular stroke therapy [122, 210]. ANSTROKE [211] and SIESTA [212, 213] have both recently demonstrated no advantage in neurological status at 24 h or 3 months for patients undergoing MT with conscious sedation.

#### Posterior circulation stroke

AIS in the posterior circulation is associated with poor prognosis with standard medical therapy (IV-tPA) [214, 215]. Basilar artery occlusion (BAO) is a form of posterior circulation stroke that results in higher rates of poor functional outcome and mortality if not recanalized [214, 216]. A systematic analysis of 420 BAO patients treated with IVT (76) and IAT (344) found that without recanalization only 2% patients were likely to have good outcome [216]. Recanalization was more common in IAT-treated patients than those who received IVT (65% (225/344) vs. 53% (40/76). The success of recanalization of acute BAO following IVT depends on thrombus length [45]. Another analysis on 592 BAO patients drawn from a prospective register also challenged the notion of unequivocal superiority of IAT over IV-tPA in BAO patients [217]. Interestingly, bridge therapies combining IAT using modern MT devices and IV-tPA have yielded good recanalization and improved survival rates for acute BAO [218–220]. Based on these findings, a multicenter randomized controlled trial, Basilar Artery International Cooperation Study (BASICS), was conceived, and is currently underway, to evaluate the efficacy of IAT plus standard medical treatment versus standard therapy alone in patients with acute BAO stroke [221, 222]. Recent studies have shown MT with the Solitaire FR device is associated with high recanalization rates and favourable outcome [223, 224].

#### Minimising the delay to endovascular reperfusion

Direct Transfer to an Endovascular Center Compared to Transfer to the Closest Stroke Centre in Acute Stroke Patients With Suspected Large Vessel Occlusion (RACECAT) is a prospective, randomized controlled trial which aims to evaluate the effectiveness of direct transfer to an endovascular stroke center based on identification of suspected large vessel occlusion in AIS patients using a prehospital screening tool, rapid artery occlusion evaluation (RACE) scale, in comparison to the transfer to the closest local stroke center [225]. The motivation behind this trial is to minimise the time to endovascular reperfusion. Triaging of AIS patients for their eligibility to IAT requires CT for determination of the presence of large artery occlusion. However, it may be possible to select patients based on non-imaging scales that can be easily applied without extensive training. Staff with emergency medical services (EMS) could be trained to use these scales (e.g., NIHSS, RACE) in screening AIS patients for their eligibility to receive reperfusion therapy.

In addition to the above issues, other treatment options such as the use of antithrombotic medications along with reperfusion therapy are also being explored. A phase III trial, Multi-Arm Optimisation of Stroke Thrombolysis [226], is investigating the efficacy of IV delivery of Argatroban and Eptifibatide in combination with rtPA in AIS. This strategy may be extended to patients who undergo MT. Some trials are currently underway or are in the planning phase to address the issues that limit the efficacy of endovascular reperfusion. A comprehensive list of ongoing and future trials is shown in Table 5.

**Table 5:** List of ongoing and upcoming trials aimed to address the issues concerning the endovascular treatment of acute ischemic stroke

Trial	Time window	Purpose	Inclusion criteria	Outcome measure
RACECAT [225]	0-8 h	Triage of the acute LVO on direct transfer to EVT-SC bypassing LSC vs. transfer to the LSC according to the current stroke protocol.	Premorbid mRS 0–2 Age ≥ 18 Suspected LVO AIS identified by a RACE scale score > 4 evaluated by EMS professional Time to arrival at EVT-SC <7 h from symptom onset	mRS at 90 days (shift analysis) Mortality at 90 days Mortality in haemorrhagic stroke patients Clinical deterioration (≥4 points on the NIHSS) Clinical benefit of direct vs. local transfer Dramatic early favourable response (NIHSS improvement ≥8 or NIHSS score of 0–2 at 24 h)
DEFUSE 3 [198]	6-16 h	Benefit of carefully selected patients with target mismatch and MCA (M1 segment) or ICA occlusion using CT/MR within 6–16 h treated with MT plus standard therapy vs. standard therapy alone.	Age 18–90 years Baseline NIHSSS is ≥6 Time to endovascular treatment since symptom onset = 6–16 h Premorbid mRS 0–2 ICA or MCA-M1 occlusion by MRA or CTA and target mismatch profile on CTP or MRI (ischemic core volume < 70 mL, mismatch ratio >/= 1.8, and mismatch volume >/= 15 mL) ASPECT on NCCT >/=6 No evidence of tumour, mass effect with midline shift, ICA or aortic dissection No occlusions in multiple vascular territories	Distribution of mRS scores at 90 days Proportion of patients with mRS 0–2 Infarct growth within 24 h Reperfusion rates at 24 h Ischemic lesion growth at 24 h
DAWN [119, 200]	6-24 h	MT using the Trevo Retriever with medical management is superior to medical management alone in improving clinical outcomes at 90 days in appropriately selected wake up and late presenting AIS within 6–24 h after symptom onset [119]	Subjects with failed IV-tPA or contraindicated for IV-tPA Age ≥ 18 Baseline NIHSS ≥ 10 Can be randomised within 6–24 h of stroke onset Pre-stroke mRS 0 or 1 <1/3 MCA territory involved, as evidenced by CT or MRI Intracranial ICA and/or MCA-M1 occlusion on MRA/CTA CIM defined on MR-DWI or CTP-rCBF maps: (a) 0- < 21 cm^3^ core infarct and NIHSS ≥10 (and age ≥ 80 years old), (b) 0- < 31 cm^3^ core infarct and NIHSS ≥10 (and age < 80 years old), or (c) 31 cm^3^to <51 cm^3^ core infarct and NIHSS ≥20 (and age < 80 years old) ICH or differential diagnosis on CT/MRI	Weighted mRS at 90 days Mortality at 90 days Good functional outcome (mRS 0–2) Revascularization rate at 24 h on CTA/MRA Neurological deterioration defined by as ≥4-point increase in the NIHSS score from the baseline score at 5–7 days or at discharge.
POSITIVE [199]	6-12 h	To determine the safety and efficacy of IAT in AIS patients Ineligible for or refractory to IV-rtPA as selected by physiologic imaging	Age ≥ 18 NIHSS ≥8 at the time of neuroimaging Time to the groin puncture 6–12 h Large vessel proximal occlusion (distal ICA through MCA M1 bifurcation) Patients who have had IV-tPA without improvement in symptoms Pre-stroke morbidity mRS 0–1 Presence of large penumbra No evidence of SAH or ICH or mass effect with midline shift <1/3 MCA territory involved, as evidenced by baseline CT or ASPECTS of >7	90 day mRS Good functional outcome mRS 0–2 at 90 days Mortality at 30 and 90 days ICH with neurological deterioration (NIHSS worsening >4) within 24 h Arterial revascularization measured by TICI 2b or 3 following MT
ENDOSTROKE [287]	NR	Predictors of the good or poor clinical outcome following MT in AIS	Age ≥ 18 years Proximal arterial vessel occlusion No evidence of venous occlusion	mRS at 90 days Complete recanalization defined by TIMI grade two or 3. Periprocedural complication rate (sICH defined by ECASS PH1 and PH2, SAH and thromboembolic events).
START [288]	0-8 h	Efficacy of the Penumbra System in AIS with a known core infarct volume at admission presenting within 8 h of onset. To study the correlation between infarct-volume and functional outcome at 90 days in MT treated patients	Age 18 to 85 years NIHSS ≥10 at admission Evidence of proximal large vessel occlusion (supra clinoid segment of ICA through the M1 segment of MCA) Patients presenting within 8 h, and those within 3 h must be ineligible or refractory to IV-rtPA Core infarct volume assessed by CTP, CTA or DWI scans within 60 min to arterial puncture. No history of stroke within 3 months No evidence of mass effect with midline shift or ICH on NCCT No evidence of arterial stenosis proximal to the occlusion that could prevent thrombectomy No evidence of preexisting arterial injury Life expectancy <90 days	Good functional outcome mRS 0–2 at 90 days Recanalization assessment using TIMI and mTICI immediately after MT Periprocedural serious events Good neurological recovery (NIHSS ≥10) at discharge Incidence of sICH and asymptomatic haemorrhage defined by ECASS criteria and patient neurological status within 24 h of the procedure.
EASI [289]	0-5 h	To evaluate the efficacy of IV-rtPA vs combined (MT plus IV-rtPA) treatment in AIS	Age ≥ 18 NIHSS ≥8 Onset to treatment less than 5 h or symptom/imaging mismatch Occlusion of MCA (m1 or M2), supraclinoid ICA or basilar trunk No evidence of haemorrhagic transformation of the infarcted territory	Favourable clinical outcome (mRS 0–2 at 90 days) sICH on CT at 24 h Infarct evolution on CT between pre-treatment and 24 h using the ASPECT score Recanalization using TICI scale after thrombectomy Procedural complication within 3 months ICH on NCCT at 24 h
BASICS [221, 222]	0-6 h	Efficacy and safety of IAT plus standard medical therapy vs. standard medical alone in patients with an acute symptomatic basilar artery occlusion (BAO)	Symptoms of BAO stroke BAO confirmed by CTA or MRA Age ≥ 18 NIHSS ≥8 at the time of neuroimaging IAT initiated within 6 h of onset of symptoms Premorbid score of 0–2 No ICH or mass effect on CT	Favourable outcome mRS 0–2 Excellent outcome mRS 0–3 Recanalization at 24 ± 6 h on CTA Volume of infarction on NCCT and CTA source images at 24 ± 6 h sICH at 24 ± 6 h on CTA Mortality at 90 days NIHSS pre, and port IV-tPA and at 24 h EQ-5D – Quality of life at 90 and 120 days
SIESTA [207, 213]	NR	Efficacy of conscious sedation vs. general anaesthesia during IAT. Update: No advantage for the use of conscious sedation recently reported [213].	Age ≥ 18 years Acute stroke in anterior circulation ICA or MCA occlusion on CTA No evidence of ICH	Higher NIHSS of >10 at 24 h NIHSS improvement mRS at 90 days Mortality before discharge or at 90 days Duration of hospital stay Recanalization status on TICI Periinterventional complications
GOLIATH [208]	NR	Efficacy of general vs. local anaesthesia during IAT	Age ≥ 18 years NIHSS > 10 mRS = <2 groin puncture < 6 h from stroke onset occlusion of ICA, ICA-T, M1, M2 GCS > 9 No evidence of posterior circulation stroke	Growth of DWI lesion (48–72 h] mRS score at 90 days Blood pressure during intervention (1–2 h) Time from arrival to the groin puncture and recanalization (1–2 h)
ANSTROKE [209]	NR	To study the efficacy of general anaesthesia vs sedation technique during embolectomy for AIS stroke (systolic pressure 140–180 mmHg)	Age ≥ 18 years CTA confirmed occlusion in ICA, ACA (A1 segment), MCA (M1 or M2 segments) NIHSS ≥14 for patients with embolus in left hemisphere or NIHSS ≥10 for embolus in right hemisphere No evidence of posterior circulation embolus No evidence of ICH on CT Pre-stroke mRS of ≤3 No evidence of spontaneous recanalization	90 day mRS Change in NIHSS score compared to admission (Day 3, 7 and 90) Degree of recanalization and reperfusion (1 day after embolectomy) Periprocedural complications Infarction magnitude CT day 1 including CTP MR on day 3 (2–4) and 3 months Brain markers (GFAP, Tau, S-100B) before, 2, 24, 48, 72 h and 3 months after the embolectomy Quantitative EEG 1, 2 and 90 days after onset Length of hospital stay Preprocedural time consumption (stroke onset to CTA, CTA to start of anaesthesia/ sedation, stroke onset to start of embolectomy and duration of embolectomy.
MOST [226]	NR	Phase III trial to explore the efficacy of IV delivery of antithrombotic medications Argatroban and Eptifibatide in combination with rtPA in AIS.	NIHSS > 6	mRS at 90 days longitudinal model relating 30 day mRS to 90 days mRS

*RACECAT* Direct Transfer to an Endovascular Centre Compared to Transfer to the Closest Stroke Centre in Acute Stroke Patients With Suspected Large Vessel Occlusion; *RACE* scale Rapid Arterial occlusion Evaluation; *mRS* Modified Rankin score; *EMS* Emergency medical service; *LVO* Large vessel occlusion; *AIS* Acute ischemic stroke; *LSC* Local stroke centre; *EVT-LSC* Endovascular stroke centre; *DEFUSE-3* Endovascular Therapy Following Imaging Evaluation for Ischemic Stroke 3; *DAWN* Trevo and Medical Management Versus Medical Management Alone in Wake Up and Late Presenting Strokes; *BASICS* Basilar Artery International Cooperation Study; *IAT* Intra-arterial therapy; *IV-rtPA* Intra venous tissue plasminogen activator; *NCCT* Non-contrast computed tomography; *CTA CT* angiography; *sICH* Symptomatic intracranial haemorrhage; *SAH* Sub-arachnoid haemorrhage; *BAO* Basilar artery occlusion; *SIESTA* Sedation vs. Intubation for Endovascular Stroke Treatment; *GOLIATH* The General or Local Anaesthesia in Intra-arterial Therapy; *MOST* The Multi-Arm Optimization of Stroke Thrombolysis; *NR* Not required; *ANSTROKE* Sedation Versus General Anaesthesia for Endovascular Therapy in Acute Stroke - Impact on Neurological Outcome; *ENDOSTROKE* International Multicenter Registry for Mechanical Recanalization Procedures in Acute Stroke; *TIMI* Thrombolysis in Myocardial Infarction; *START* Imaging Guided Patient Selection for Interventional Revascularization Therapy; *EASI* Endovascular Acute Stroke Intervention Trial

### Conclusions, discussions, and future recommendations

**Fig. 2:**
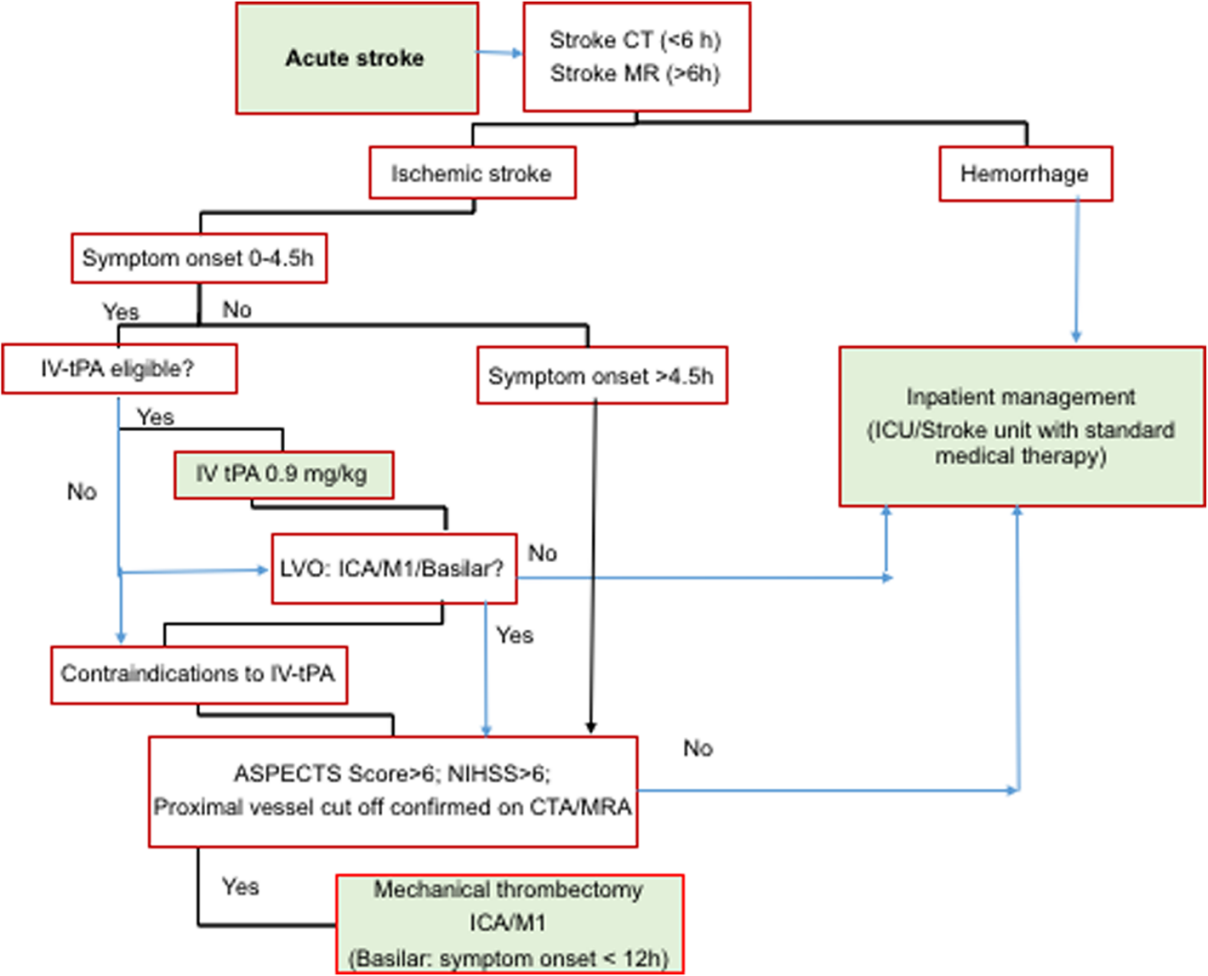
Workflow diagram detailing the algorithm to select AIS patients with or without large artery occlusion for IV-tPA, MT, or IV-tPA followed by MT. CT = computed tomography; MRI = magnetic resonance imaging; IV-tPA = intravenous trans-plasminogen activator; ICU = intensive care unit; CTA = CT angiography; MRA = MR angiography; LVO = large vessel occlusion; NIHSS = National Institutes of Health Stroke Scale; ICA = internal carotid artery

The approved treatment for acute ischemic stroke is reperfusion therapy using systemic thrombolysis or endovascular mechanical thrombectomy. The recent success of endovascular trials has revolutionised the way large artery occlusion stroke patients are managed. AHA/ASA has accordingly updated its guidelines for stroke care. The recommendation is to use MT with stent retriever in combination with standard therapy (IV-rtPA) in AIS patients, aged ≥18 years; baseline ASPECTS score ≥ 6, baseline NIHSS score ≥ 6 with angiographically confirmed large vessel occlusion presenting within 6 h of symptom onset (Level 1A evidence) [227, 228]. The workflow algorithm detailing the standard of care for IV-tPA and mechanical thrombectomy in AIS patients is shown in Fig. 2 (algorithm has been updated keeping in consideration recently published DAWN study [119, 202]).

Endovascular therapy also provides an alternative to systemic thrombolysis for patients who fail to reperfuse with IV-rtPA or those who are not eligible for IVT due to restricted time-window, or those with unknown onset or “wake-up” stroke, or contraindications [153, 159, 215]. However, due to a shortage of neurointerventionists or endovascular specialists experienced in the procedure [197], procedural complexity, high costs, pre-hospital delays, inter-facility transfers, and a limited number of institutions that offer endovascular treatment, only a select number of patients will likely be offered this therapy [229]. Future efforts to shorten the endovascular procedural time, build infrastructure to provide MT, and increase access to endovascular facilities are required [27]. Comprehensive stroke centers with access to IV and IA techniques, trained stroke neurointerventionists, imaging-guided treatment workflow, and access to advanced neurosurgical support will pave the way for high-end stroke care delivery. Meanwhile, given the present limitations, careful selection of patients is crucial to maximize the gain for appropriate patients. It has been suggested that patients with angiographically confirmed proximal large vessel occlusion with a viable penumbra on MRI and NIHSS > 18, within 8 and 24 h of stroke onset in the anterior and posterior circulation respectively, should be given endovascular MMRT [159]. Other authors recommend a lower threshold of NIHSS ≥7 on the addition of advanced neuroimaging parameters including ASPECTS ≥6 or 7, and angiographically confirmed large vessel occlusion with moderate-to-good collaterals on multiphase CT to select candidates for endovascular MT using stent retriever [230]. With regards to MRI led patient selection based on infarct volume, endovascular therapy is also preferred for large artery occluded AIS patients with small infarct defined by early DWI volume < 70 mL, and an accessible proximal occlusion. Endovascular MMRT or standalone MT is not suitable with distal occlusion or lacunar stroke. High revascularization yields obtained using (a) MMRT or combinational approaches that include IA/IV thrombolytic agents, stenting, angioplasty, aspiration, clot retrieval, (b) second generation MT devices such as Solitaire and Trevo, (c) combined MT interventions using suction embolectomy with large bore microcatheters such as Penumbra MAX [104–107] and stent retrievers, and (d) with latest generation Penumbra 3D separator [100, 108, 109] are encouraging.

In conclusion, both IV thrombolysis and endovascular treatment have been incorporated into the standard of care in stroke therapy. In light of recent studies in favour of imaging based selection of AIS patients for reperfusion therapy, the time window of MT may extend to 24 h (or beyond) since symptom onset [119, 202]. However, further research, exploring bridge therapy or MMRT in addition to advanced imaging-based approaches to select appropriate patients, may also widen the time-window for patient selection and would contribute immensely to early thrombolytic strategies, better recanalization rates, and improved clinical outcomes.

## Abbreviations

AHA/ASA: American Heart Association/American Stroke Association
AIS: Acute Ischemic Stroke
ANSTROKE: Sedation Versus General Anaesthesia for Endovascular Therapy in Acute Stroke
ASPECTS: Alberta Stroke Program Early CT Score
ATLANTIS: Alteplase Thrombolysis for Acute Noninterventional Therapy in Ischemic Stroke
BAO: Basilar artery occlusion
BASICS: Basilar Artery International Cooperation Study
CASES: Canadian Alteplase for Stroke Effectiveness Study
CBV: Cerebral blood volume
CTA: CT angiography
CTP: CT Perfusion
DAWN: DWI or CTP Assessment with Clinical Mismatch in the Triage of Wake-Up and Late Presenting Strokes Undergoing Neurointervention
DEFUSE: Diffusion and Perfusion Imaging Evaluation for Understanding Stroke Evolution
DVT: deep venous thrombosis
ECASS: European Collaborative Acute Stroke Study
EMS: Emergency medical services
EPITHET: Echoplanar Imaging Thrombolysis Evaluation Trial
ESCAPE: Endovascular Treatment for Small Core and Anterior Circulation Proximal Occlusion with Emphasis on Minimizing CT to Recanalization Times
ESCAPE: Endovascular Treatment for Small Core and Proximal Occlusion Ischemic Stroke
ESO: European Stroke Organisation
EXTEND-IA: Extending the Time for Thrombolysis in Emergency Neurological Deficits–Intra-arterial
FDA: Federal Drugs Agency
GA: General anaesthesia
GOLIATH: General or Local Anaesthesia in Intra-arterial Therapy
HERMES: Highly Effective Reperfusion evaluated in Multiple Endovascular Stroke Trials
IAT: Intra-arterial thrombolysis
ICA: Internal carotid artery occlusion
ICH: Intra-cerebral hemorrhage
IMS: Interventional Management of Stroke
IST: International Stroke Thrombolysis
IV rtPA: Intravenous recombinant tissue plasminogen activator
MCA: Middle cerebral artery
MELT: Middle Cerebral Artery Embolism Local Fibrinolytic Intervention Trial
MMRT: Multimodal reperfusion therapy
MR CLEAN: Multicenter Randomized Clinical Trial of Endovascular Treatment for Acute Ischemic Stroke in the Netherlands
MRA: Magnetic resonance angiography
mRS: Modified Rankin Score
MT: Mechanical thrombectomy
NCCT: non-contrast computed tomography
NIHSS: National Institutes of Health Stroke Scale
NINDS: National Institute of Neurological Disorders and Stroke
NNT: Number need to treat
PAI: Plasminogen activator inhibitor
POSITIVE: Perfusion Imaging Selection of Ischemic Stroke Patients for Endovascular Therapy
PROACT: Prolyse in Acute Cerebral Thromboembolism
RACE: Rapid artery occlusion evaluation
RACECAT: Direct Transfer to an Endovascular Centre Compared to Transfer to the Closest Stroke Centre in Acute Stroke Patients with Suspected Large Vessel Occlusion
REVASCAT: Randomized Trial of Revascularization with Solitaire FR Device vs Best Medical Therapy in the Treatment of Acute Stroke Due to Anterior Circulation Large Vessel Occlusion Presenting within 8 h of Symptom Onset
SARIS: Stent-Assisted Recanalization in Acute Ischemic Stroke
SES: Self-expanding stents
sICH: Symptomatic intracerebral haemorrhage
SIESTA: Sedation vs Intubation for Endovascular Stroke Treatment
SITS-ISTR: Safe Implementation of Thrombolysis in Stroke-International Stroke Thrombolysis Register
SK: Streptokinase
START: Imaging-Guided Patient Selection for Interventional Revascularization Therapy
SWIFT PRIME: Solitaire with the Intention for Thrombectomy as Primary Endovascular Treatment of Acute Ischemic Stroke
SYNTHESIS: Local Versus Systemic Thrombolysis for Acute Ischemic Stroke
TAFI: Thrombin-activatable fibrinolysis inhibitor
THERAPY: Assess the Penumbra System in the Treatment of Acute Stroke
THRACE: Trial and Cost-Effectiveness Evaluation of Intra-arterial Thrombectomy in Acute Ischemic Stroke
TICI: Thrombolysis in Cerebral Infarction
TIMI: Thrombolysis in Myocardial Infarction
TK: Tenecteplase
TTP: Time to peak
UK: Urokinase
uPA: urinary-type plasminogen activator
